# Using the MCF10A/MCF10CA1a Breast Cancer Progression Cell Line Model to Investigate the Effect of Active, Mutant Forms of EGFR in Breast Cancer Development and Treatment Using Gefitinib

**DOI:** 10.1371/journal.pone.0125232

**Published:** 2015-05-13

**Authors:** Darrell C. Bessette, Erik Tilch, Tatjana Seidens, Michael C. J. Quinn, Adrian P. Wiegmans, Wei Shi, Sibylle Cocciardi, Amy McCart-Reed, Jodi M. Saunus, Peter T. Simpson, Sean M. Grimmond, Sunil R. Lakhani, Kum Kum Khanna, Nic Waddell, Fares Al-Ejeh, Georgia Chenevix-Trench

**Affiliations:** 1 QIMR Berghofer Medical Research Institute, Brisbane, Queensland, Australia; 2 Queensland Centre for Medical Genomics, The Institute for Molecular Bioscience, University of Queensland, Brisbane, Queensland, Australia; 3 The University of Queensland, UQ Centre for Clinical Research, Brisbane, Queensland, Australia; 4 The University of Queensland School of Medicine, Brisbane, Queensland, Australia; 5 Pathology Queensland, The Royal Brisbane & Women’s Hospital, Brisbane, Queensland, Australia; Seoul National University, REPUBLIC OF KOREA

## Abstract

**Background:**

Basal-like and triple negative breast cancer (TNBC) share common molecular features, poor prognosis and a propensity for metastasis to the brain. Amplification of *epidermal growth factor receptor* (*EGFR*) occurs in ~50% of basal-like breast cancer, and mutations in the epidermal growth factor receptor (*EGFR*) have been reported in up to ~ 10% of Asian TNBC patients. In non-small cell lung cancer several different mutations in the EGFR tyrosine kinase domain confer sensitivity to receptor tyrosine kinase inhibitors, but the tumourigenic potential of EGFR mutations in breast cells and their potential for targeted therapy is unknown.

**Materials and Methods:**

Constructs containing wild type, G719S or E746-A750 deletion mutant forms of *EGFR* were transfected into the MCF10A breast cells and their tumorigenic derivative, MCF10CA1a. The effects of EGFR over-expression and mutation on proliferation, migration, invasion, response to gefitinib, and tumour formation *in vivo* was investigated. Copy number analysis and whole exome sequencing of the MCF10A and MCF10CA1a cell lines were also performed.

**Results:**

Mutant EGFR increased MCF10A and MCF10CA1a proliferation and MCF10A gefitinib sensitivity. The EGFR-E746-A750 deletion increased MCF10CA1a cell migration and invasion, and greatly increased MCF10CA1a xenograft tumour formation and growth. Compared to MCF10A cells, MCF10CA1a cells exhibited large regions of gain on chromosomes 3 and 9, deletion on chromosome 7, and mutations in many genes implicated in cancer.

**Conclusions:**

Mutant EGFR enhances the oncogenic properties of MCF10A cell line, and increases sensitivity to gefitinib. Although the addition of EGFR E746-A750 renders the MCF10CA1a cells more tumourigenic *in vivo* it is not accompanied by increased gefitinib sensitivity, perhaps due to additional mutations, including the *PIK3CA* H1047R mutation, that the MCF10CA1a cell line has acquired. Screening TNBC/basal-like breast cancer for *EGFR* mutations may prove useful for directing therapy but, as in non-small cell lung cancer, accompanying mutations in *PIK3CA* may confer gefitinib resistance.

## Introduction

Breast cancer is the most common cancer in women and the second most common cause of cancer death, after lung cancer, in women in Australia (http://www.aihw.gov.au/). The most aggressive forms of breast cancer are triple negative breast cancer (TNBC), defined histologically by the absence of estrogen receptor (ER), progesterone receptor (PR) and epidermal growth factor 2 (HER2), and a subset of TNBC referred to as basal-like breast cancer, characterized by CK5/6 and/or epidermal growth factor receptor (EGFR) expression [1–3]. Both tumour types are associated with shorter disease-free and overall survival, propensity for lung and brain metastases, younger age at diagnosis, African-American descent and lack of response to endocrine or HER2-mediated therapies [4–12]. There is no targeted therapy available for these tumour types so new tools to evaluate TNBC/basal-like breast cancer are required to improve prognostic capability and to predict response to standard chemotherapy.

Mutations in the tyrosine kinase domain of epidermal growth factor receptor 1 (*EGFR*) occur in ~10–40% non-small cell lung cancers (NSCLC), with the highest incidence in patients of Asian descent [13–16]. Deletions in exon 19 and in-frame missense mutations in exon 21 account for ~90% of these mutations with other mutations in exon 18, such as G719S, accounting for the rest [17]. Several of the most common mutations found in NSCLC, including G719S or E746-A750, result in the activation of EGFR pathways and oncogenic transformation [18, 19]. Tumours harbouring these *EGFR* mutations are more sensitive to tyrosine kinase inhibitors (TKI) that target EGFR, such as gefitinib, erlotinib or cetuximab [20, 21]. Several phase III clinical trials have reported improved progression-free survival (PFS) in NSCLC patients harbouring *EGFR* mutations who are treated with gefitinib or erlotinib compared to those treated with standard chemotherapy [22–27].

More recently, mutations in *EGFR* have been identified in TNBC in up to ~11% (8/70) of Asian patients [28], although these mutations seem much rarer in European and Australian breast cancer cases, at 1.3% (3/229) and 0% (0/50), respectively [29, 30]. However, *EGFR* mutations have also been found in 1/12 brain metastases from breast and 3/9 metastases from other primary cancers, suggesting that activation of the EGFR pathway may play a role in the metastatic development of breast cancer [20]. One of the downstream modulators of EGFR signalling *PIK3CA*, exhibits activating mutations in brain metastases from breast (2/12) and other cancers (2/9) [20]. Further, *EGFR* copy number gain, or *PTEN* loss or *PIK3CA* mutation have been shown to promote brain metastases from breast cancer [31]. As TKIs have been found to improve progression free survival (PFS) in NSCLC patients, determining the consequences of these EGFR mutations in breast cancer could be of benefit to shaping the management of disease.

MCF10A is a spontaneously immortalized, non-malignant breast cell line obtained from a patient with benign fibrocystic disease [32] and is the founder cell line of a progressively more aggressive family of breast cancer lines. These cell lines include MCF10AT1 (MCF10AT), a premalignant cell line derived from MCF10A transfected with H-Ras [33], and a set of oncogenic MCF10CA cell lines (including MCF10CA1a), which gained a *PIK3CA* H1047R activating mutation after *in vivo* passage of MCF10AT [34]. While MCF10A cells are incapable of forming tumours, MCF10AT can form tumours with an incidence of about 25% [33] and MCF10CA1a always forms tumours after subcutaneous injection into nude mice [34]. The MCF10 cell line series therefore provides a useful model to assess the oncogenic potential of genes of interest. We used the MCF10A and MCF10CA1a cell lines to assess the role of the common E746-A750 deletion (*EGFR-DEL*) mutation, and the less oncogenic *EGFR* G719S missense mutation, in promoting oncogenesis and gefitinib resistance in breast cells.

## Materials and Methods

### Ethics Statement

This study was conducted in strict accordance with the guidelines in the current National Health and Medical Research Council Australian Code of Practice for the Care and Use of Animals for Scientific Purposes (*Code of Practice*), the Queensland Animal Care and Protection Act 2001 and the accompanying Animal Care and Protection Regulation 2002, and the relevant Federal and State legislation and regulations. This research was covered by Animal Ethics Committee approval from the QIMR Berghofer Medical Research Institute. All surgery was performed under 2.5% isofluorane/oxygen anesthetic, and all efforts were made to minimize suffering.

### Cell lines and reagents

MCF10A (ATCC, Manassas, VA) and MCF10CA1a (gift from Pauley Robert from the Barbara Ann Karmanos Cancer Institute, Detroit, Michigan) cells were maintained in DMEM/F12 (Invitrogen, Carlsbad, CA) supplemented with 5% horse serum (Invitrogen), 500ng/ml hydrocortisone (Sigma-Aldrich, St. Louis, MO), 100ng/ml cholera toxin (Sigma-Aldrich), 10 μg/ml insulin (Invitrogen) and 20ng/ml epidermal growth factor (EGF, Sigma-Aldrich) (“full media”). Some assays were performed in reduced (2%) serum media in the presence or absence of EGF. Gefitinib, afatinib, docetaxol and doxorubicin were purchased from Selleck Chemicals (Houston, TX) and maintained as a 10 mM stock in dimethyl sulphoxide (DMSO). Mouse anti-β-actin, mouse anti-α-tubulin and rabbit anti-mouse HRP-conjugate antibodies were obtained from Sigma-Aldrich, mouse anti-EGFR monoclonal antibody (clone 225) was isolated from hybridoma cultures (HB-8508 from the ATCC) as previously described [35], Alexa488 conjugated rabbit anti-mouse antibody was obtained from Invitrogen, and IRDye 800CW goat anti-mouse antibody was obtained from Millenium Science (Mulgrave, Australia). Growth factor-reduced Matrigel was obtained from Invitrogen.

### Transfections

The retroviral vector pBABEpuro containing either *EGFR*-WT, *EGFR*-G719S (gifts from H. Greulich), or *EGFR*-E746-A750del or no insert (empty vector) (all confirmed by sequencing) were transfected into the amphotropic packaging cell line, Phoenix (ATCC), using Lipofectamine 2000 (Invitrogen). After 48 h viral supernatant was harvested, filtered (45 μm) and used to infect MCF10A cells in the presence of 8 μg/mL polybrene (Sigma-Aldrich). Cell lines were selected with 1 μg/mL puromycin for one week (Sigma-Aldrich) and designated MCF10A *EGFR*-WT, MCF10A *EGFR*-GS and MCF10A *EGFR*-DEL, respectively. To aid in assessing tumour formation assays, MCF10A and MCF10CA1a cells were tagged with luciferase using the MSCV Luciferase PGK-hygromycin (Addgene) retroviral vector. Vector DNA was transfected into Phoenix cells as described above. Virus was harvested 48, 54, 72 and 78 post-transfection and used in sequential infection of the MCF10CA1a cells (total of 4 infections) with 8 μg/ml polybrene. Stable transfectants were selected with the addition of 200 μg/ml hygromycin (Sigma-Aldrich). The luciferase-tagged cell lines were then infected with the *EGFR*-WT, *EGFR*-G719S, *EGFR*-E746-A750del vectors and designated MCF10CA1a- *EGFR*-WT, *EGFR*-GS or *EGFR*-DEL, accordingly. MCF10CA1a cells infected with empty pBABEpuro retroviral vector (EV) were used as controls (MCF10A and MCF10CA1a-EV).

### Western blotting

Three to four million cells were lysed in modified radio immunoprecipitation assay (RIPA) buffer (25 mM Tris HCl pH 7.6, 151.5 mM NaCl, 1% NP-40, 1% sodium deoxycholate, 0.1% SDS, 1x Complete mini protease inhibitor (Roche, Indianapolis, IN), 1 mM PMSF) for 40 minutes on ice. The lysates were clarified by centrifugation (15 minutes at 14000 rpm). 30–40 μg of lysate was resolved by SDS-PAGE and transferred onto polyvinylidene difluoride membranes (Immobilon, Millipore, Billerica, MA). Membranes were blocked with either 5% skim milk diluted in PBS-Tween (0.1%) or Odyssey blocking buffer (Licor Biosciences, Lincoln, NE) then incubated with anti-EGFR, anti-β-actin or anti-α-tubulin diluted in blocking buffer at 4°C overnight. The filters were incubated with secondary antibodies conjugated to horse radish peroxidase and the immunocomplexes detected with the Chemiluminescence HRP Substrate kit (Millipore) or incubated with IRDye conjugated secondary antibodies and the immunocomplexes detected by fluorescence using the Licor Odyssey Classic (Licor Biosciences).

### Proliferation

Cells were seeded at a density 4000, 2000, 1000 or 500 cells per well in triplicate in 96 well plates, incubated at 37°C and proliferation monitored with the MTS (3-(4,5-dimethylthiazol-2-yl)-5-(3-carboxymethoxyphenyl)-2-(4-sulfophenyl)-2H-tetrazolium, inner salt) assay (Promega, Madison, WI) or using real-time imaging with the Incucyte ZOOM (Ann Arbor, MI, USA). Briefly, for the MTS assay, at the designated time points MTS was diluted 1:10 in growth media to a final concentration of 0.5 mg/ml and 100 μl added to each well. The plate was incubated for 4 hours at 37°C, the reaction stopped with the addition of SDS to a final concentration of 1% and absorbance at 490 nm read. For the Incucyte, phase contrast images of the cells were obtained every 6 hours and confluency determined using the Incucyte ZOOM software.

### Drug response

Cells were seeded at a density of 4000 cells/well in duplicate or triplicate in 96 well plates and incubated overnight at 37°C in the full media with or without EGF (20 ng/ml). After 24 hours, the media was replaced with media (with or without EGF) containing serial dilutions of gefitinib. Concentrations from 0.49–4000 nM were used for the MCF10A lines and from 7.8–125 nM for the MCF10CA1a lines in assays using the Incucyte Zoom to measure cell viability, and from 15.625–1000 nM in MTS assays. Cell viability was monitored with the MTS assay for the MCF10A cell lines as described for cell proliferation. The Incucyte ZOOM was used to monitor cell viability for the MCF10CA1a lines by monitoring cell confluency as described for cell proliferation and by monitoring the increase in dead cells. For the latter, 1–2 drops/ml of NucGreen Dead 488 Ready Probes reagent (Invitrogen) was added to the gefitinib-containing media and green fluorescent images of the cells were obtained every 6 hours with the phase contrast images. The number of green objects representing dead cells was counted using the Incucyte ZOOM software. MTS assays were also performed on the MCF10CA1a lines using serial dilutions from 1000 nM to 15.625 nM of gefitinib or afatinib or from 1000 μg/ml to 15.625 μg/ml cetuximab. Cells were seeded at 1000 cells/well in triplicate in a 96 well plate incubated overnight at 37°C in full media with EGF then washed and treated with the above drug concentrations in full media without EGF and incubated for seven days. Data was analysed in GraphPad Prism Version 6.05 (GraphPad Software, La Jolla, CA, USA) using nonlinear regression of normalized inhibitor responses and automatic outlier detection.

### Combination therapy assay

The MCF10CA1a cell lines were seeded at 1000 cells/well in triplicate in a 96 well plate and incubated overnight at 37°C in full media. The next day the cells were washed and incubated in full media without EGF containing either serial dilutions of chemotherapy alone (docetaxel/doxorubicin at a 5:1 ratio—a ratio determined to minimize drug dose and maximize *in vivo* responses in a mouse model of triple negative breast cancer [35]) or chemotherapy with afatinib at its calculated ½ IC50 concentations. Cell survival was measured using the MTS assay after seven days of treatment. The concentrations used for the chemotherapy treatment were (docetaxel/doxorubicin) 10 nM/2 nM, 5/1, 2.5/0.5, 1.25/0.25, 0.625/0.125, 0.3125/0.0625, 0.15625/0.03125. For afatinib the doses of used were 41 nM (MCF10CA1a), 35 nM (MCF10CA1a-*EGFR*-WT), 34 nM (MCF10CA1a-*EGFR*-GS), and 29 nM (MCF10CA1a-*EGFR*-DEL), respectively.

### Acini formation assays

Cells were resuspended at 1.25x10^4^ cells/ml in assay media containing 2% growth factor reduced Matrigel with or without EGF (5 ng/ml), 400 μl added to 8-well chamber slides (Thermo-Scientific, Waltham, MA) pre-coated with 60 μl growth factor reduced Matrigel and incubated at 37°C. Phase contrast images were obtained on day 13 using the 4X objective of an Axiovert 135 microscope (Zeiss, Jena, Germany) and reviewed using ImageJ 1.44p software [36]. After 14 days the acini were fixed in methanol at -20°C, blocked in 1% bovine serum albumin (BSA) in phosphate buffered saline (PBS), stained for α-tubulin diluted in 1% BSA in PBS and counterstained with DAPI (Sigma-Aldrich) in PBS-Tween 20 (0.1%). The chambers were dismantled, Prolong Gold antifade (Invitrogen) was added and coverslips were affixed to the slides. Confocal fluorescent images were taken with a Leica TCS SP2 confocal microscope (Leica Microsystems, North Ryde, Australia) using the Leica Confocal software.

### Migration and invasion

For migration assays, cells were seeded in duplicate into the top chamber of a 16-well RTCA CIM Plate 16 for the xCELLigence system for real-time cellular analysis (John Morris Scientific, AUS) in serum-free media (SFM) at a density of 4x10^4^ cells/well. Media containing 20 ng/ml EGF was aliquoted into the bottom chamber and the migration assessed by measuring electrical impedene every 5 minutes for up to 78 hours. Invasion assays were performed in a similar fashion except 30 μl of a 1:10 dilution of growth factor reduced Matrigel in SFM was aliquoted into the top chamber of each well and allowed to set at 37°C for 3–4 hours. Cells were seeded in duplicate at a density of 1x10^5^ cells/well and invasion monitored as described above.

### Tumour formation

MCF10A and MCF10ACA1a were infected with either *EGFR*-wild type (WT), *EGFR*-G719S (GS), *EGFR*-E746-A750del (DEL) or empty vector (EV) as described above. Exponential growth phase cells (5x10^6^) were collected and prepared in a 1:1 (v:v) solution of PBS/Matrigel for injection into the mammary fat pads of female BALB/c nude mice at 5–6 weeks of age. Tumour growth was monitored with bioluminescent live luciferase imaging (BLI) or calliper measurements. Tumours were surgically resected after reaching 250 mm^3^ in volume (calculated as: shortest diameter^2^ x longest diameter/2) and mice were further monitored for metastatic deposits by BLI.

### Genomic DNA preparation

Genomic DNA from the cell lines was extracted using the DNeasy Blood & Tissues Kit (Qiagen, Chadstone, Vic, Australia) according to manufacturer’s instructions. Briefly, cells were cultured to 80–90% confluency in T-75 flasks, trypsinized, washed in PBS and lysed using following the standard protocol. The DNA was eluted in 200 μl of the provided elution buffer and concentration determined by measuring absorbance at 260 nm on the NanoDrop 2000. gDNAs were stored at -20°C until used for exome sequencing and SNP-chip assays below.

### Copy number analysis by SNP array

DNA (200ng) from the MCF10A and MCF10CA1a cell lines was analyzed for copy number alterations using Illumina Human Omni 2.5M SNP arrays (2.5–8 v1.1). Copy number was quantified and summarized using GAP [37], and copy number changes across all chromosomes for a given sample were visualized by Circos plots [38]. Copy number was classified as follows: 0 = deletion, 1 = loss, 3–5 = medium gain, and 6–8 = high level gain. The raw data was submitted to the Gene Expression Omnibus (GEO) database (accession number: GSE59800).

### Exome sequencing

Exome sequencing libraries were generated using the Nextera Rapid Capture Exome (Illumina, San Diego, CA). Fifty ng of gDNA was used to generate a whole genome-sequencing library. Sequence libraries were subsequently pooled into sets of 12 prior to hybridization and enrichment. Note that all hybridizations, washes and enrichments were performed using the manufacturer’s recommendation. In all cases, libraries at both the whole genome and enriched exome stages were qualified and quantified using the DNA High sensitivity assay (BioAnalyzer 2100 Agilent). Exome sequencing was performed using the Illumina 2500 HiSEQ platform. Cluster generation and sequencing was completed using TruSeq PE Cluster Kit v3-cBot-HS and TruSeq SBS Kit v3-HS (200 cycle) kits respectively with libraries clustered at 8pM using the cBOT instrument (Illumina). Three sequencing reads were generated including 101bp paired-end sequencing reads and a single index read which allows for de-multiplexing of the library pools. Clustering and sequencing were performed following the manufacturer’s recommendations.

### Variant calling and prediction

Sequencing data was aligned to the human genome (hg19) using BWA [39]. Cell line specific variants were identified using qSNP [40] (heuristic driven somatic/germline caller) and the Genome Analysis Tool Kit (GATK) [41] (a Bayesian caller). Only variants that were called by both qSNP and GATK were used in subsequent analyses. The Pindel tool [42] was used to identify insertion-deletion events. Cell line specific variants were subsequently annotated for gene consequence using ENSEMBL v70.

Several online tools were used to determine the effects of the rare variants discovered by exome sequencing. Either the nucleotide and/or the amino acid residue changes were entered into the PolyPhen-2 [43] and Provean [44] online software tools. The Provean online tool uses both the Provean and SIFT [45–49] algorithms to determine the pathogenicity of the mutations. The Ingenuity Pathway Analysis tool was used to assess the gene lists for potentially damaging rare variants in each line (IPA, QIAGEN Redwood City, www.qiagen.com/ingenuity).

## Results

### EGFR activating mutations mediate EGF-independent proliferation in MCF10A

**Fig 1:**
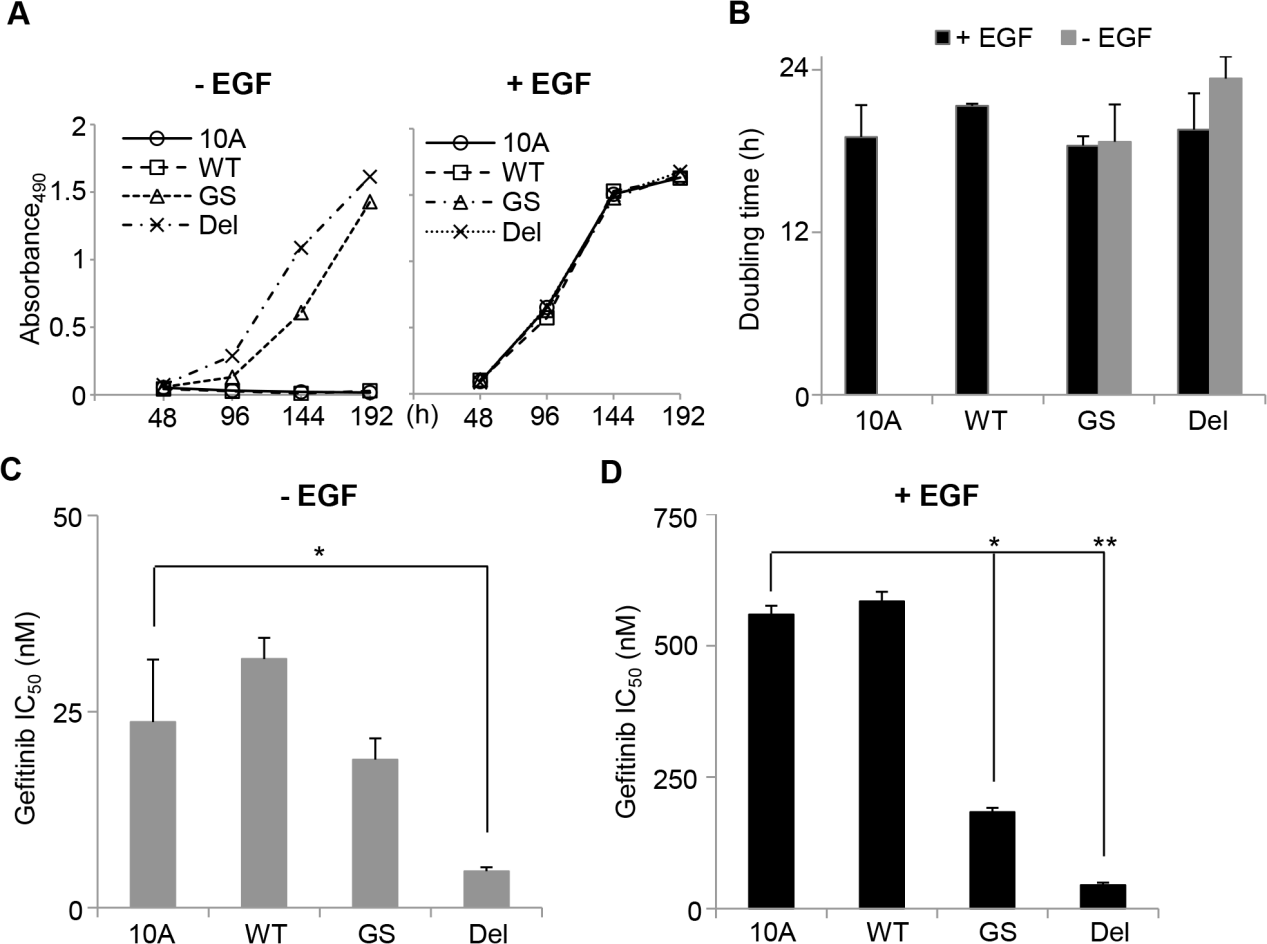
Expression of the *EGFR*-GS and—DEL mutant proteins enhances MCF10A EGF-independent growth and sensitivity to gefitinib. **A.** Cells were cultured in the presence (+ EGF) or absence (- EGF) of EGF and their proliferation assessed using MTS assay by measuring absorbance at 490 nm over a period of 8 days. **B.** The doubling times of the cell lines were calculated from the rates of proliferation. As neither MCF10A nor MCF10A-WT cell proliferated in the absence of EGF, a doubling time could not be calculated. **C.** Cells were treated with varying concentrations of gefitinib in the absence of EGF for 72 hours and cell viability measured using the MTS assay. The IC_50_ was calculated and the differences compared using the unpaired t-Test (* p<0.05). **D.** Cells were treated with varying concentrations of gefitinib in the presence of EGF for 72 hours and cell viability measured using the MTS assay. The IC_50_ was calculated and the differences compared using the unpaired t-Test (**** p<0.0001). 10A: MCF10A-EV, WT: MCF10A-*EGFR*-WT, GS: MCF10A-*EGFR*-GS, DEL: MCF10A-*EGFR*-DEL. Data from duplicate experiments.

As triple negative breast cancers (TNBC) have been found to harbour mutations in *EGFR* [28], we wished to determine how they affect a relatively normal breast cell line. We used MCF10A cells, a spontaneously immortalized, non-malignant cell line obtained from a patient with fibrocystic disease [32], and stably infected them with *EGFR*-wild type (WT), *EGFR*-G719S (GS) or *EGFR*-DEL (E746-A750) (DEL) retroviral constructs. All three constructs expressed EGFR well *in vitro* (S1 Fig). The proliferation of the MCF10A cell lines containing the *EGFR* constructs was monitored in the presence or absence of EGF (Fig 1A and 1B). Neither wild type *EGFR*, nor *EGFR* mutant expression, affected the proliferation of the MCF10A cells in the presence of EGF. However, in the absence of EGF both *EGFR*-GS and *EGFR*-DEL mutant expression resulted in increased proliferation of the MCF10A cells compared to the parental control, or the cells expressing wild type EGFR.

### MCF10A cells expressing activated EGFR mutations are sensitive to the EGFR inhibitor, gefitinib

Several different somatic EGFR mutations have been shown to confer sensitivity to tyrosine kinase inhibition (TKI) in cancer models [50]. To determine whether the *EGFR*-GS or *EGFR*-DEL mutants could affect MCF10A sensitivity to TKIs, survival was monitored in the presence of a range of gefitinib concentrations in media with or without EGF (Fig 1C and 1D). MCF10A cells displayed a significant sensitization to gefitinib in EGF-free media (IC_50_ 24±8 compared to 560±20 nM in EGF containing media, p<0.0001). Over-expression of wild type EGFR did not alter the response of MCF10A to gefitinib IC_50_ (EGF-free media: 32±3 nM; EGF containing media: 580±20 nM). In contrast, expression of either the GS or DEL mutant EGFR increased the sensitivity of MCF10A cells in media containing EGF (183±8 nM, p<5x10^-6^; 45±5 nM, p<1x10^-6^, respectively). The MCF10A *EGFR*-DEL cells, but not the MCF10A *EGFR*-GS cells (19±3 nM) were also more sensitive to gefitinib in EGF-free media (4.7±0.5 nM, p<0.05).

### Mutant EGFR promotes spheroid formation in MCF10A cells

**Fig 2:**
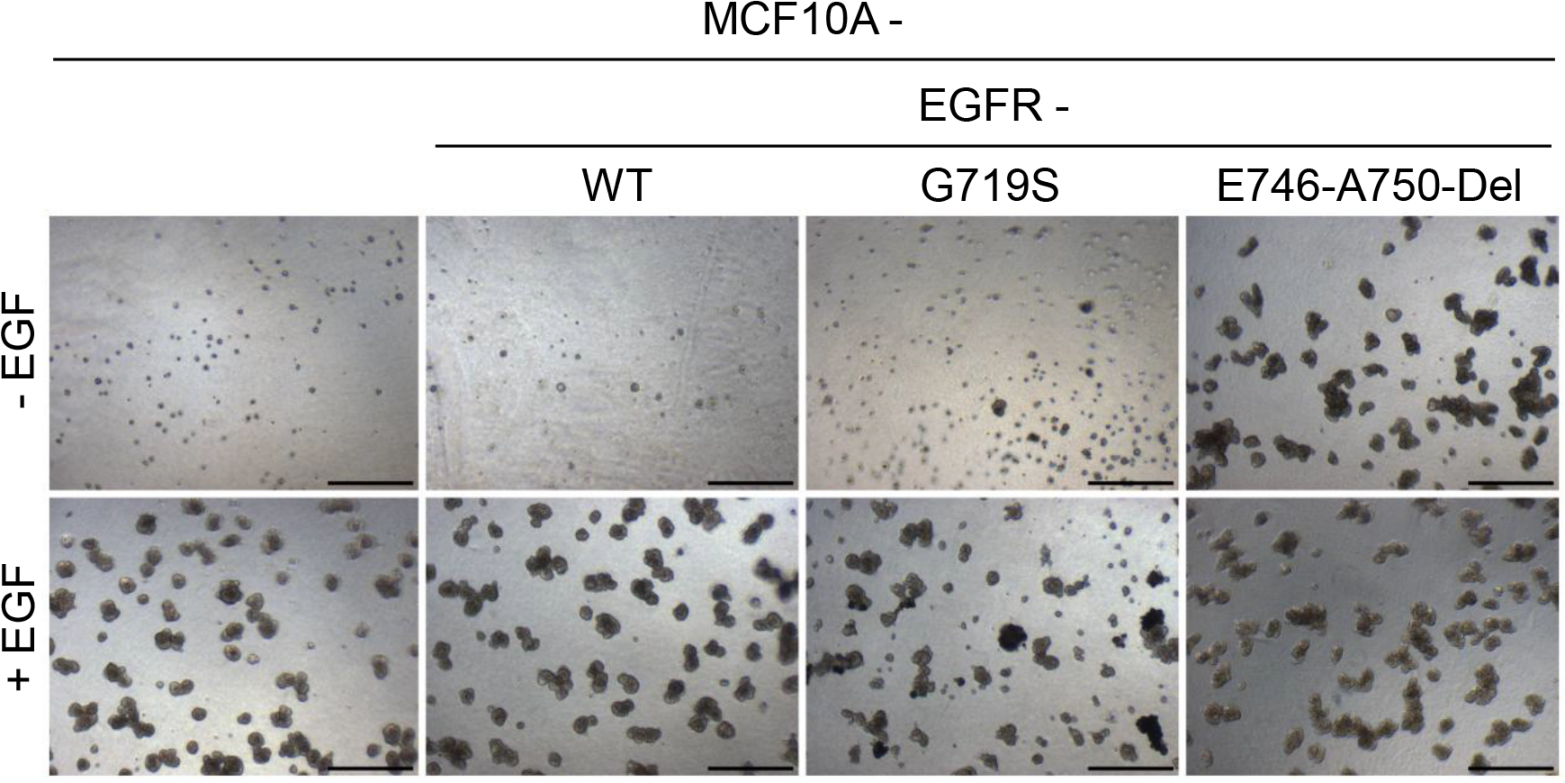
*EGFR*-DEL expression enhances anchorage-independent growth of MCF10A cells in the absence of EGF. Cells were cultured in media containing growth factor-reduced Matrigel with 2% horse serum in the absence (- EGF) or presence of 5 ng/ml EGF (+ EGF). At 13 days spheroid formation assessed was assessed with phase contrast microscopy. Scale bars = 400 μm.

Microscopic analysis of two dimensional culturing of the EGFR cell line derivatives revealed only minimal differences in cell morphology (S2 Fig) so acini formation was assessed to determine whether expression of *EGFR*-GS or *EGFR*-DEL mutants can alter acini morphology, as a marker of the oncogenic potential of MCF10A cells [51]. In the presence of EGF all four cell lines produced acinar structures of 50–100 μm size, though the acini formed by the MCF10A *EGFR*-GS cells tended to be more heterogenous and slightly dispersed (Fig 2). MCF10A formed acini with mostly empty luminal spaces but the acini formed by MCF10A *EGFR*-WT cells in the presence of EGF were also partially occluded (S3 Fig). In the absence of EGF, only MCF10A *EGFR*-DEL cells formed acini at a rate similar to that seen in the presence of EGF. MCF10A *EGFR*-GS cells formed some acini in the absence of EGF, but these were small and infrequent; MCF10A and MCF10A *EGFR*-WT cells did not form acinar structures in the absence of EGF (Fig 2). The luminal spaces of the acini formed by the MCF10A *EGFR*-GS and—DEL cells were partially occluded by cells in either the presence or absence of EGF (S3 Fig). These results suggest that expression of the *EGFR*-DEL mutant may increase the oncogenic potential of MCF10A cells.

### Expression of EGFR-DEL increases tumourigenicity of MCF10CA1a but not MCF10A cells

**Fig 3:**
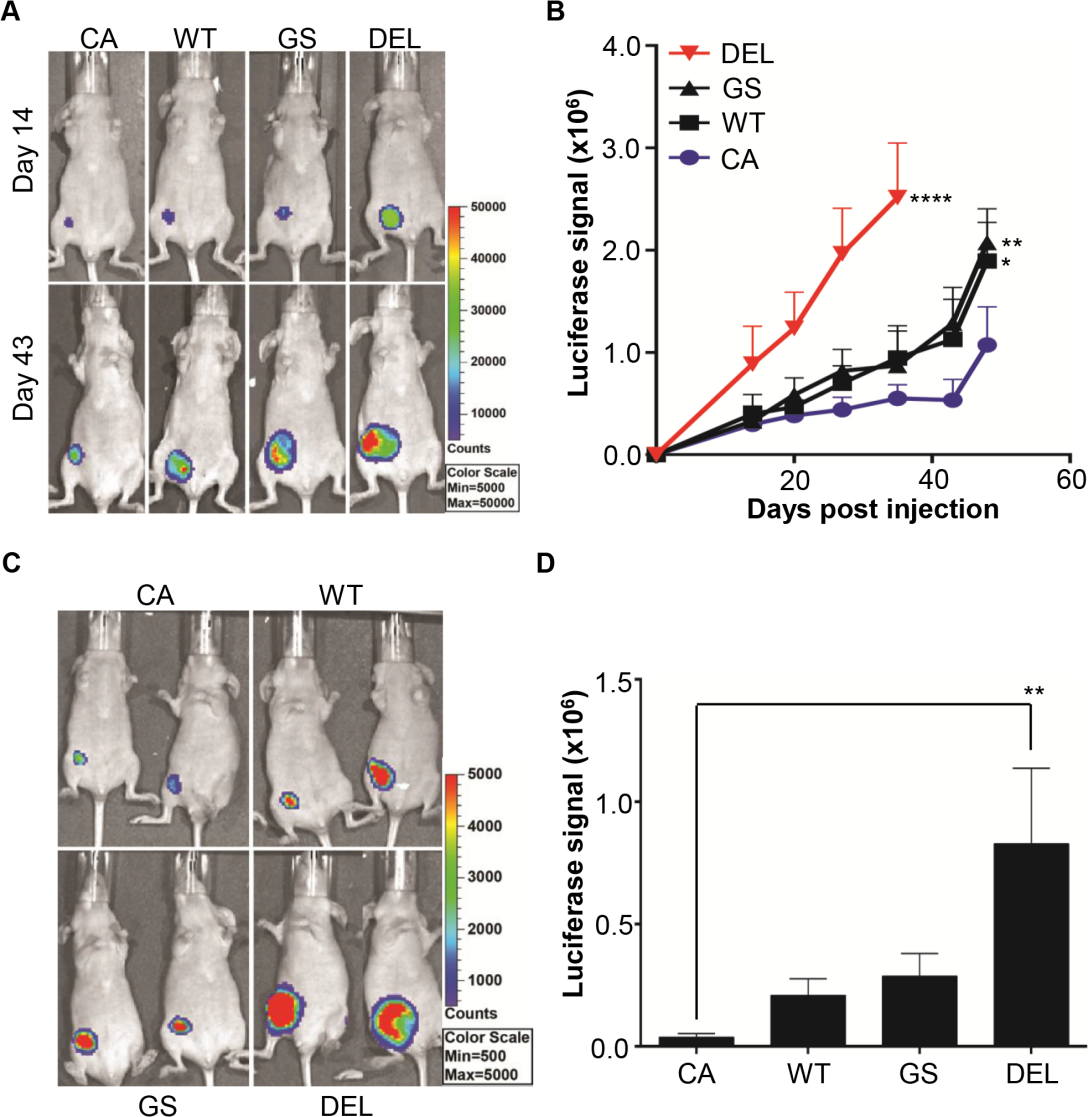
Overexpression of wild type or mutant *EGFR* increases the growth of MCF10CA1a mammary fat pad xeongrafts. Female BALB/c nude mice were injected in the mammary fat pad with 5x10^6^ cells from the indicated cell line and tumour formation was monitored with bioluminescent imaging. CA: MCF10CA1a-EV, WT: MCF10CA1a -*EGFR*-WT, GS: MCF10CA1a -*EGFR*-GS, DEL: MCF10CA1a -*EGFR*-DEL **A.** Representative bioluminescent images of individual mice taken on day 14 and day 43. **B.** Plot of the increase in luciferase signal in each group of mice (*p<0.05, **p<0.01, ****p<0.0001, n = 5, vs CA control, One-Way ANOVA). **C.** Representative bioluminescent images of individual mice taken on day 49 post injection in an independent cohort of mice. **D.** Plot of the magnitude of luciferase signal in each group of mice at day 49 post injection (**p<0.01, n = 5).

*EGFR*-DEL and *EGFR*-GS expression in MCF10A cells resulted in an increased proliferative ability *in vitro* and *EGFR*-DEL expression could induce spheroid formation in media lacking EGF. To determine whether these changes were reflected by increased tumorigenicity *in vivo* we examined the growth of mammary fat pad xenografts of the various MCF10A EGFR cell lines. The expression of the *EGFR* constructs in the MCF10A cells did not induce primary tumour formation up to 6 months post-injection (data not shown). As the MCF10A cell line is not malignant and the *EGFR* mutants were not sufficient to induce tumourigenesis, we decided to use the MCF10A derivative cell line, MCF10CA1a. MCF10CA1a is a fully malignant cell line that gained a spontaneous *PIK3CA* H1047R activating mutation [34] after *in vivo* passaging of MCF10AT cells, which were derived by transfecting active mutant *HRAS* G12V into MCF10A cells [33]. After generating a stable luciferase-expressing MCF10CA1a cell line, the cell line was engineered to express our *EGFR* constructs with an empty vector (EV) serving as a control (S1 Fig). The growth of the tumours induced by the MCF10CA1a cells was significantly increased by the expression of *EGFR*-DEL and to a lesser extent by *EGFR*-GS and *EGFR*-WT expression compared to the MCF10CA1a-EV cells (Fig 3).

The primary tumours from the MCF10CA1a-*EGFR*-DEL and—GS expressing cells were surgically resected (6 weeks post injection) and the animals monitored for metastasis using bioluminescence of the tumours. Luciferase signals were only observed in the MCF10CA1a-*EGFR*-DEL tumour-resected mice (2 out 5) but were lost after 6 months (S4 Fig). Similar results were observed in an independent experiment where signals were initially observed outside of the primary tumour site but disappeared after 6 months (data not shown). These luciferase signals may not be indicative of true metastatic deposits, but, rather, may be dormant deposits that failed to overcome natural killer cell activity which is known to be increased in adult nude and other athymic strains of mice compared to mice with intact immune systems [52–54].

### EGFR mutant expression increases EGF-independent proliferation of MCF10CA1a cells but has little effect on EGFR tyrosine kinase inhibitor sensitivity

**Fig 4:**
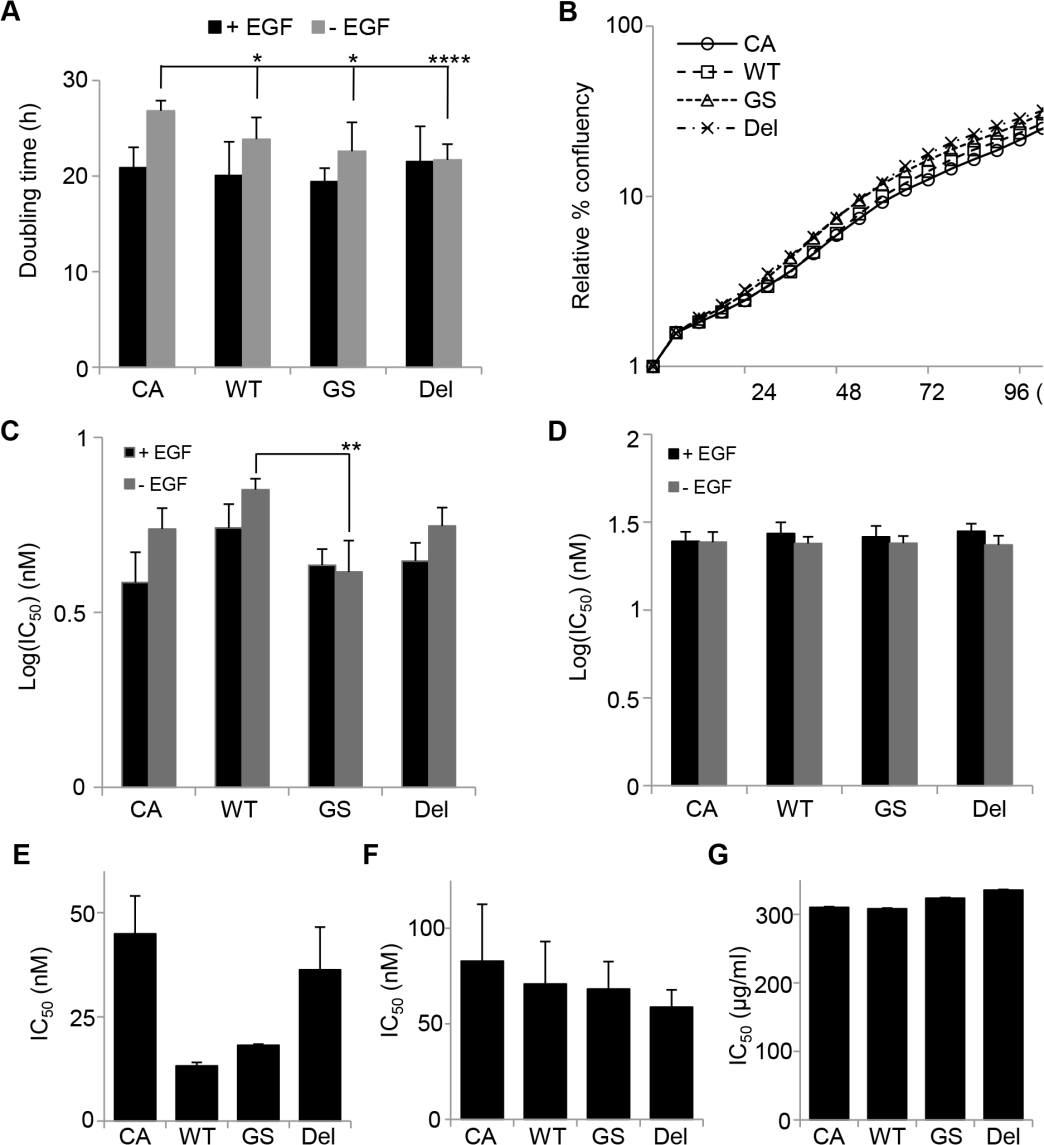
Expression of the *EGFR*-GS and—DEL mutant proteins enhances MCF10CA1a EGF-independent growth but not sensitivity to gefitinib. **A.** Cells were cultured in the presence (+ EGF) or absence (- EGF) of EGF and their proliferation assessed using real-time imaging on the Incucyte Zoom and measuring the increase in confluency over 4–5 days. The doubling times of the cell lines were calculated from the rates of proliferation from 36–60 hours (24 hour period). While there was no difference in proliferation between the cell lines in the presence of EGF, in the absence of EGF all three *EGFR* constructs reduced MCF10CA1a doubling time (*p<0.05, **** p<0.0001^,^, unpaired T-test, n = 6). **B.** Representative growth curves for cells grown in EGF-free media with measurements of confluency taken every 6 hours for up to 102 hours. **C-D.** Calculated IC_50_ values of gefitinib for inhibition of proliferation (**C**) or cell toxicity (**D**) from cells treated for 48 hours in various doses of gefitinib and monitored in real-time using the Incucyte Zoom (** p<0.01, unpaired T-test, n = 3). **E**-**G.** Calculated mean IC_50_ values from cells treated for seven days in various doses of gefitinib (**E**, n = 2), afatinib (**F**, n = 4) or cetuximab (**G**, n = 1) and cell death determined using the MTS assay. CA: MCF10CA1a-EV, WT: MCF10CA1a-*EGFR*-WT, GS: MCF10CA1a-*EGFR*-GS, DEL: MCF10CA1a-*EGFR*-DEL.

We found that MCF10CA1a cells expressing mutant *EGFR* were the more aggressive compared to EV-control cells in the *in vivo* model of tumour formation. We therefore performed further characterization *in vitro* of MCF10CA1a cells expressing wildtype or mutant *EGFR*. *EGFR*-WT (doubling time: 23.87±2.27 h, p<0.05), *EGFR*-GS (22.59±3.04 h, p<0.05) and *EGFR*-DEL (21.7±1.65 h, p<5x10^-4^) expressing MCF10CA1a cells demonstrated a faster proliferation rate than the MCF10CA1a–EV control cells (26.84±1.06 h) in EGF-free media (Fig 4A and 4B). Treatment with high concentrations of gefitinib in the presence or absence of EGF inhibits proliferation and induces cell death (S5 Fig) in all the MCF10CA1a lines. Interestingly, none of the *EGFR* constructs affected MCF10CA1a cell proliferation sensitivity (Fig 4C) or cell death response to gefitinib in the short term Incucyte assays (Fig 4D) but both *EGFR*-WT and *EGFR*-GS constructs but not EGFR-DEL exhibited a trend for decreased MCF10CA1a cell survival compared to the parental cells as measured by the long term MTS assay (Fig 4E). Two other EGFR inhibitors, afatinib and the monoclonal antibody cetuximab, also induce MCF10CA1a cell death independently of wild type or mutant EGFR expression (Fig 4F and 4G).

**Fig 5:**
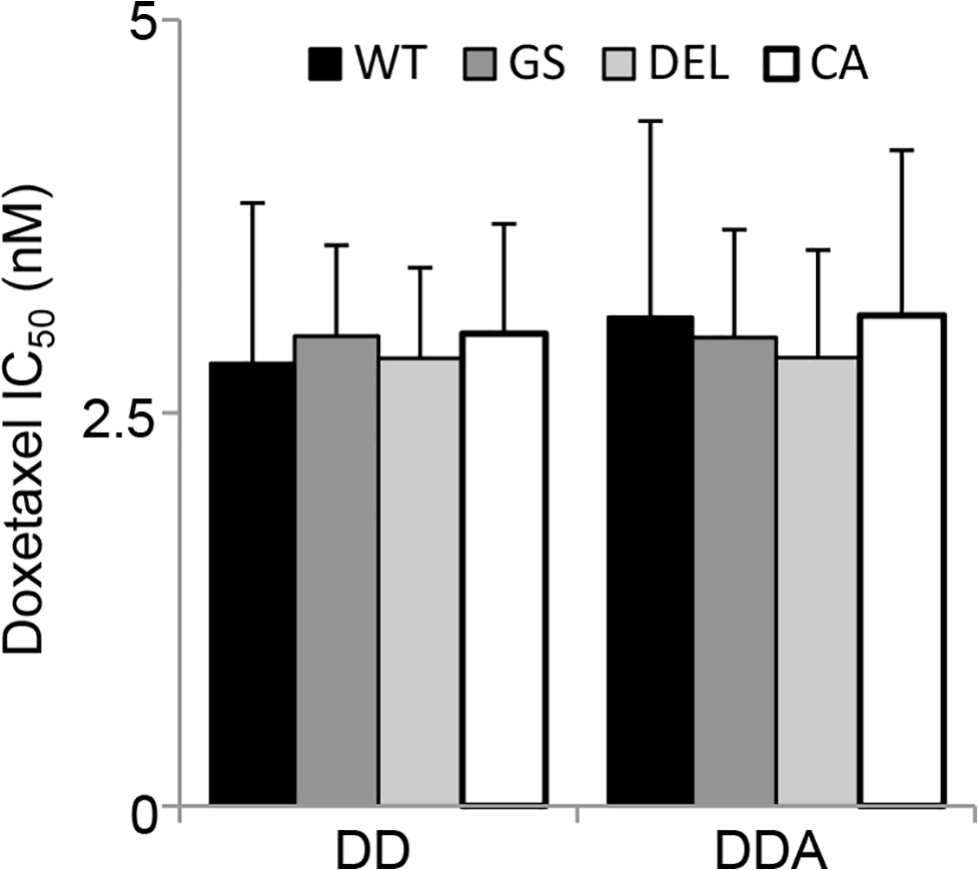
Docetaxel/doxorubicin chemotherapy of MCF10CA1a is unaffected by *EGFR* mutation or in combination with afatinib. Cells were cultured in the presence of EGF and treated with docetaxel/doxorubicin in a 5:1 ratio (DD) alone or in combination with the half IC_50_ dose of afatinib (DDA) for seven days. Cell survival was assessed using the MTS assay and the IC_50_ dose was reported using the docetaxel concentrations. CA: MCF10CA1a-EV, WT: MCF10CA1a-*EGFR*-WT, GS: MCF10CA1a-*EGFR*-GS, DEL: MCF10CA1a-*EGFR*-DEL.

To determine if the presence of the EGFR mutants would alter MCF10CA1a cell sensitivity to standard anthracycline/taxane-based chemotherapy we treated cells with various concentrations of the docetaxel/doxorubicin dose regimen previously used in our lab [35] combined with the half IC_50_ dose of afatinib for each cell line (Fig 5, S6 Fig **)**. The addition of afatinib or gefitinib (data not shown) to docetaxel/doxorubicin did not alter the sensitivity of the any of the cell lines to the chemotherapy regime.

### EGFR-GS and EGFR-DEL expression increases the rate of migration and invasion of MCF10CA1a cells

**Fig 6:**
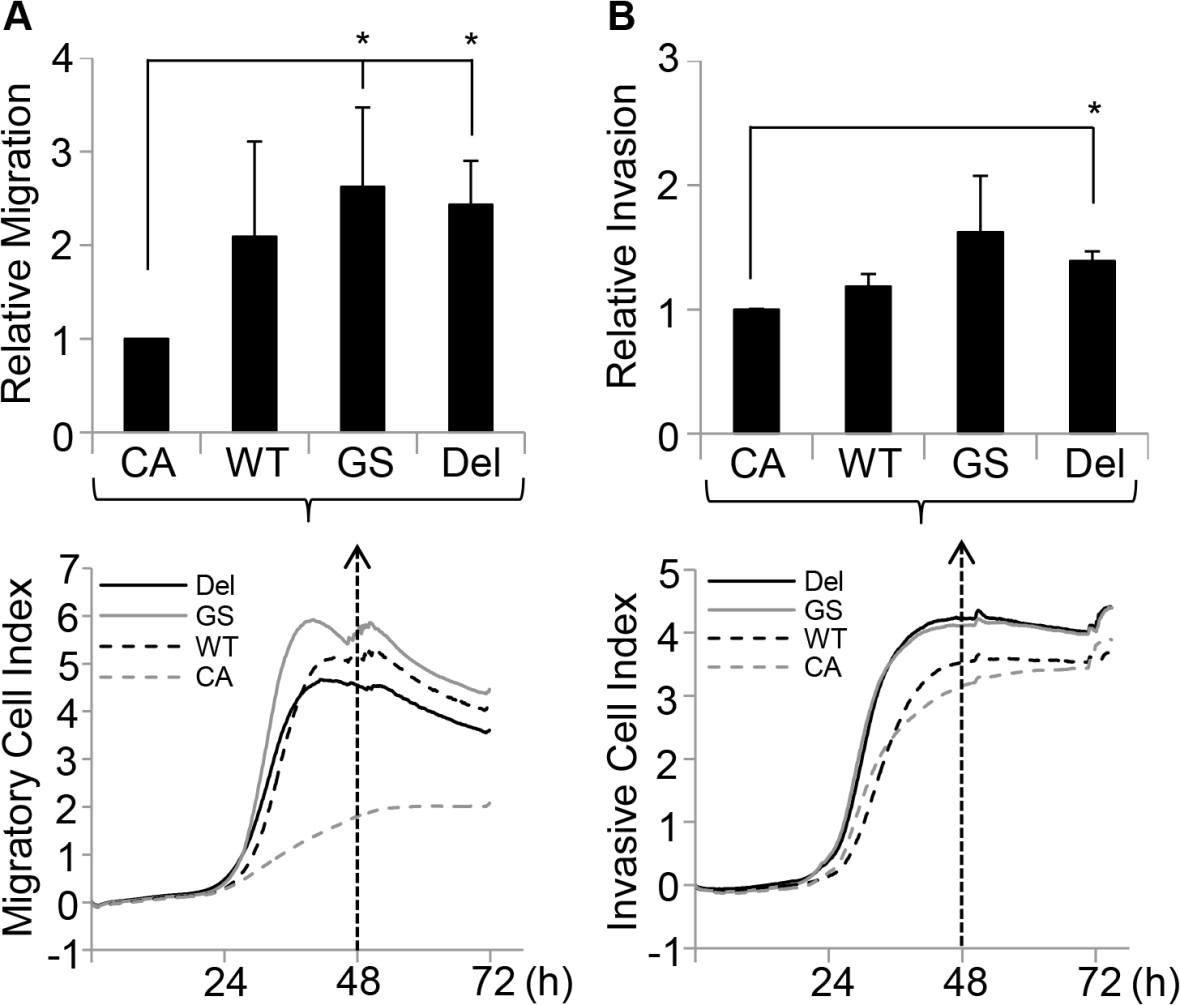
Mutant EGFR expression increases the migratory and invasive abilities of MCF10CA1a cells. **A.** MCF10CA1a cells expressing empty vector (CA), wild-type *EGFR* (WT), G719S *EGFR* (GS) or E746-A750 Deletion *EGFR* (DEL) were seeded in serum-free media (SFM) at 40000 cells/well in 16-well xCELLigence plates and their migration to full media containing 20 ng/ml EGF monitored. The top panel is the average of the relative migration of the cells at 48 hours of three independent experiments while the bottom panel is a representative trace from one experiment of the absolute migration index over the course of the experiment (*p<0.05, unpaired T-test). **B**. MCF10CA1a cells expressing empty vector (CA), wild-type *EGFR* (WT), G719S *EGFR* (GS) or E746-A750 Deletion *EGFR* (DEL) were seeded in SFM containing Matrigel at 100000 cells/well in 16-well xCELLigence plates and their invasion to full media containing 20 ng/ml EGF monitored. The top panel is the average of the relative invasion of the cells at 48 hours of two independent experiments while the bottom panel is a representative trace from one experiment of the absolute invasion index over the course of the experiment (*p<0.05, unpaired T-test).

A hallmark of cancer is the increased ability of cancer cells to migrate and invade through the extracellular matrix [55]. Migration and invasion of the MCF10CA1a cells containing the EGFR constructs was assessed using the xCelligence real-time monitoring system. Both MCF10CA1a *EGFR*-GS and *EGFR*-DEL cells migrated faster than the control empty vector cells (relative migration: 2.62±0.85, p< 0.05; 2.43±0.47, p<0.05) to media containing 5% horse serum and 20ng/ml EGF after 48 hours **(** Fig 6A **)**. The MCF10CA1a *EGFR*-DEL cells could invade Matrigel 1.39±0.08 times faster than empty vector control cells at 48 hours (p<0.05) in media containing 5% horse serum and 20ng/ml EGF (Fig 6B). These results suggest that *EGFR*-DEL and, to a lesser extent, EGFR-G719S mutations may confer metastatic properties on breast cancer cells. This was observed to some extent with the ability of the MCF10CA1A *EGFR-DEL* cells injected into mice to temporarily colonize the brain and other sites after the primary tumour removal, though true metastases were not formed (S4 Fig).

### Copy number analysis and exome sequencing of MCF10A and MCF10CA1a cells

**Fig 7:**
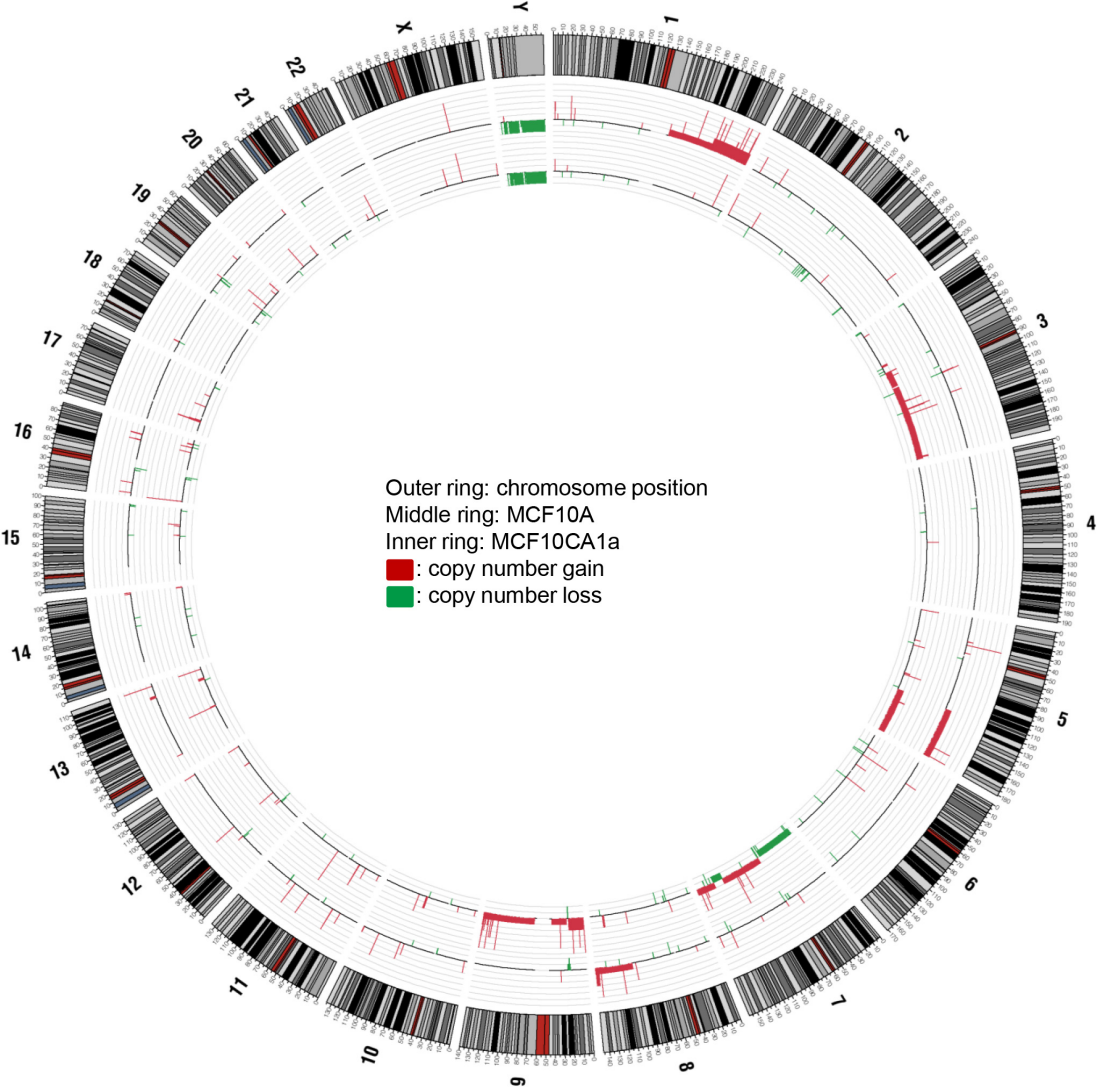
Circos plot summarizing the gene amplification and loss differences between MCF10A and MCF10CA1a. The outer band details chromosome number and position, middle band represents MCF10A and the inner band represents MCF10CA1a. In each case, values plotted are from copy number analysis of Illumina Human Omni 2.5M SNP array data. Red regions highlight gene amplification while green regions represent gene loss.

Although the exome of MCF10A has been sequenced [56], there are no published reports of the exome sequence of MCF10CA cells. We therefore sequenced the MCF10CA1a, and MCF10A cell lines, in order to determine whether additional somatic mutations in MCF10CA1a, apart from those known to have occurred in *HRAS* and *PIK3CA*, might contribute to its tumourigenicity and resistance to gefitinib. We also compared copy number variations between these two lines using Illumina Human Omni 2.5M SNP arrays. Several prominent regions of difference were found between MCF10A and MCF10CA1a, including large regions of copy number gain in chromosomes 1 and 8 in MCF10A that were missing in MCF10CA1a, and regions of gain on chromosomes 3 and 9, and regions of both gain and loss on chromosome 7 in MCF10CA1a relative to MCF10A (Fig 7). Gain of 2374 protein-coding genes and loss of 461 genes was present in MCF10A (S1 and S2 Tables) while MCF10CA1a showed gain of 1433 genes and loss in 33 genes (S2 and S3 Tables). Relative to the reference genome, gain of 544 and loss in 24 genes were found to be in common between the two cell lines (S3 Table).

We performed exome sequencing of the MCF10A and MCF10CA1a cell lines using Illumina Nextera technology and the average sequencing read depth was ~200 reads for both cell lines. The cell line specific variants were called as described and are shown in S4 and S5 Tables. The G12V *HRAS* mutation introduced into the MCF10A line to generate the precursor MCF10AT line [33] and the spontaneous H1047R *PIK3CA* mutation that arose in the MCF10CA1a line [57] were correctly identified in the MCF10CA1a cells. In total, 85 rare variants (which could be germline or somatic in origin) were identified as specific to MCF10A cells and 172 rare variants (assumed to be somatic mutations) were identified in the MCF10CA1a cells (Table 1). Of these 24/85 and 43/172 were synonymous mutations in the MCF10A and MCF10CA1a lines, respectively, with the rest potentially affecting splicing or protein coding.

**Table 1:** Frequency of each class of mutations in MCF10A and MCF10CA1a cells.

Mutation type	MCF10A (%)	MCF10CA1a (%)
Missense	47 (58.0%)	98 (55.4%)
Nonsense	2 (24.7%)	10 (5.6%)
Non-stop	0 (0.0%)	1 (0.6%)
Frameshift Deletion	2 (2.4%)	2 (1.2%)
In-frame Deletion	2 (2.4%)	1 (0.6%)
Frameshift Insertion	3 (1.7%)	0(0.0%)
Splice Site	7 (8.2%)	16 (9.3%)
Synonymous	24 (29.6%)	43 (24.3%)
**Total**	85	172

Several online tools (PolyPhen-2, Provean, SIFT) were used to assess the potential effect of the rare variants on gene function (S4 and S5 Tables). PolyPhen-2 and SIFT only provide information on missense mutations but Provean provides limited information on other non-synonymous variants as well. Approximately 50% of the missense variants in both the MCF10A (26/47) and MCF10CA1a (55/98) lines were predicted to be damaging by at least two out of three software tools; a number of these variants, or the genes in which they were found, have been linked to cancers.

We analyzed the gene lists of the potentially damaging rare variants for each line using the Ingenuity Pathway Analysis to determine the key cellular functions and diseases associated with these genes (S7 Table). Cancer was a top three disease identified by IPA in both MCF10A (top disease) and MCF10CA1a (third disease). The top molecular and cellular functions were predicted to be cellular morphology in the MCF10A list andcell death and survival in the MCF10CA1a list.

## Discussion

Herein we report the effect of activating mutations of *EGFR* on the tumourigenic properties of MCF10A and MCF10CA1a cells. While ectopic expression of active forms of *EGFR* did not affect either MCF10A or MCF10CA1a proliferation in the presence of EGF, it did enhance cell proliferation of both cell lines in the absence of EGF (Figs 1A and 1B and 4A and 4B). This was most apparent in the MCF10A line, which did not grow in media without EGF, even with expression of wild type *EGFR*. The presence of mutant *EGFR* also induced spheroid formation in the absence of EGF and led to spheroids with partially occluded luminal spaces (S3 Fig). However, the enhanced oncogenic properties of the mutant *EGFR* MCF10A cells did not lead to tumour formation in nude mice suggesting that EGFR activation is not sufficient for tumourigenicity in MCF10A cells and a second (or more) genetic alteration is required. In contrast, mutant *EGFR* expression increased invasive potential, migratory ability and the rate of xenograft tumour formation from MCF10CA1a cells significantly compared to the control empty vector or wild type *EGFR*-expressing MCF10CA1a cells. Dissemination outside the primary tumour site was observed in the xenografts containing activating EGFR mutations. However, this distant colonisation did not develop into overt distant metastases. These *in vivo* observations are in concordance with the increased proliferation, invasion and migration we observed *in vitro* with the mutant *EGFR* MCF10CA1a cells (Fig 6).

While mutant *EGFR* did enhance the ability of MCF10CA1a cells to form tumours in nude BALB/c mice, it was unable to induce metastases after resection of the primary tumours (**S4 Fig**) or after intravenous injections in lung colonization experiments. MCF10CA1a has been shown to form lung metastases when injected into the tail vein of SCID/beige mice [34], so lack of metastases in this study may be due to difference in the mouse strains used. As it is known that the natural killer (NK) cell activity is greater in nude (including BALB/c) compared to thymic mice [52–54], it is possible that the lack of metastasis formation is due to enhanced NK cell activity.

Activating mutations in *EGFR*, including the G719S and E746-A750 deletion mutants used in this study, show increased sensitivity to TKIs [20, 21], so we therefore assessed the effect of the TKI gefitinib on cell proliferation in MCF10A and MCF10CA1a cells (Figs 1C and 1D; 4C and 4D and S5 Fig). MCF10A cells displayed increased sensitivity to gefitinib when expressing mutant *EGFR*. In MCF10CA1a cells, expression of *EGFR*-WT or—GS but not—DEL displayed a increase in gefitinib sensitivity in an equivalent assay. This may be due to changes in gene expression or to different somatic mutations between the MCF10A and MCF10CA1a cell lines. It is known that activating mutations in the *HRAS* homolog, *KRAS*, decrease gefitinib sensitivity in NSCLC [58, 59], and that activation of the PIK3CA pathway also reduces tyrosine kinase inhibitor sensitivity in *EGFR*-mutant lung cancers [60–63]. Furthermore, our whole exome sequencing analysis of the MCF10CA1a cells revealed a potentially damaging Y228H mutation in *ATXN2*, a gene that has been shown to affect EGFR trafficking via endocytic internalization [64]. Interestingly, the *ATXN2* gene has been found to be altered by copy number, mutation or expression in ~9% of breast cancers in the TCGA dataset (S6 Table) and this may be another mechanism for tumour cells to potentiate EGFR signalling. No changes in TKI sensitivity was observed when the cell lines were treated with the EGFR inhibitors with or without treatment with the clinically relevant chemotherapy 5:1 docetaxel/doxorubicin.

As the MCF10CA1a cell line is an *in vivo*-selected clonal derivative of the MCF10A cell line in the progression series, we assessed the genetic variation between the two cell lines by determining copy number and substitution/indels in each cell line. MCF10A shared large regions of copy number gain with MCF10CA1a in 5q, deletions in chromosome 9, 15p, 21p and 22p, and had a unique region of copy number gain in 1q and 8q not shared by MCF10CA1a (Fig 7). Conversely, MCF10CA1a also displayed copy number gain at 3q, 7q and chromosome 9, copy number loss in 7p and 7q, and a deletion at Xp. In addition, several smaller regions of copy number gain and loss were identified in each cell line (S1 and S2 Tables). The copy number gains at 3q, 5q, 8q and chromosome 9, and the loss at 21p have been previously reported [65, 66] and several other smaller regions of copy number variation have been previously identified as well. The copy number gain at 1q in MCF10A cells, however, is unique to this study and several alterations found in previous studies were not identified in this study. This may be due to variation in the cultured cell populations and evolution of the MCF10A cell line.

Whole exome sequencing was performed on the MCF10A and MCF10CA1a parental cell lines to determine the nature of the somatic mutations that occurred in the progression from a relatively normal cell line (MCF10A) to a more aggressive, tumourigenic cell line (MCF10CA1a). The sequencing analysis identified both of the previously known mutations in the MCF10CA1a cells absent from the MCF10A line (G12V *HRAS*and H1047R *PIK3CA*). The MCF10CA1a cells harboured approximately twice the number of cell line specific mutations than the MCF10A cells (Table 1). More of the mutations in the MCF10CA1a cells predicted to be damaging have been previously linked to cancer compared to the mutations in specific to the MCF10A cells. For example, *TP53BP2*, *CDK11A*, *MME* and *SEPT6* are mutated in MCF10CA1a cells and have strong correlations to a number of cancers [67–69].

CDK11A is a cyclin-dependent kinase [70] with roles in apoptosis [71] and may act as a tumour suppressor in neuroblastoma, melanoma and non-Hodgkin’s lymphoma [72–74] but may be tumour promoting in osteosarcoma and liposarcoma [75, 76]. MME (CD10/CALLA), a matrix metalloendopeptidase, is a well known marker of B-cell acute lymphoblastic leukemia [77] and has also been linked to renal cell carcinoma [78]. SEPT6 is a member of the septin family of polymerizing GTPases required for cortical organization and cytokinesis [79] and has been implicated in leukemia [80–82]. *SEPT6* also exhibits frequent mutations or copy number variations in a number of cancers, including breast (COSMIC (http://cancer.sanger.ac.uk/cancergenome/projects/cosmic; Catalogue of somatic mutations in cancer)). *CDK11A*, *MME* and *SEPT6* were also found to altered in 6.2%, 4.5%, and 3.7%, respectively, in a panel of 1062 breast tumours from the Cancer Genome Atlas project [83, 84], either through mutation, amplification, deletion or altered gene expression.

*TP53BP2*, also known as ASPP1 (apoptosis stimulating protein of p53-2), is a tumour suppressor gene that activates the pro-apoptotic functions of p53 distinctly from its cell cycle arrest functions [85, 86]. It binds to p53 and a number of other, potentially regulatory, proteins through a highly conserved C-terminal region (reviewed in [87]). Alter expression of *TP53BP2* in human tumours has been shown in a number of cancer types, including down-regulation in breast cancer [86, 88] and lung cancer [89–91]. Reduced expression of *TP53BP2* has been linked with poor distant recurrence-free surival in breast cancer patients [88] and was observed in metastatic breast cancer samples compared to non-metastatic samples [92], suggesting that *TP53BP2* may be associated with breast cancer progression. *TP53BP2* is altered in ~26% of the breast tumours from TCGA database (S6 Table) but, paradoxically, is predominantly amplified or overexpressed (http://www.cbioportal.org/, [83, 84]). It is possible that the T838R mutation in *TP53BP2* in MCF10CA1a cells may contribute to their malignancy, perhaps by decreasing TP53BP2 protein activity.

A recent Phase II clinical trial was conducted examining the ability of the anti-EGFR monoclonal antibody cetuximab to treat metastatic TNBC patients, either alone or in combination with carboplatin [93]. Though most of the TNBCs had EGFR pathway activation, only a minority exhibited pathway inhibition after one to two weeks of inhibitor therapy, suggesting that EGFR-independent mechanisms are responsible for constitutive pathway activation. This was suggested to be due to EGFR downstream pathway activation and/or K-Ras activation. This exemplifies the need to understand tumour genetic context in influencing drug sensitivity and the importance to implement precision medicine in order to overcome these issues. The differences between MCF10A and MCF10CA1a from the CNV and exome sequencing analysis identified in this study may be useful to guide future diagnostic efforts to understand EGFR inhibition sensitivity in breast cancer.

Interestingly, a naturally occuring constitutively active variant of *EGFR* with an in-frame 801 bp deletion excising most of the extracellular domain has been found in primary breast tumours [94]. This variant has been associated with cancer stem cell characteristics and can increase tumourigenicity of human breast cancer cell lines *in vitro* and *in vivo*. Stem cell-like cells in breast cancer have been suggested to be treatment resistant populations that can promote tumour recurrence [95]. The findings of this study suggest that while EGFR activating mutations are relevant to understanding the etiology and progression of breast cancer, they are insufficient to drive chemotherapeutic resistance. Other EGFR mutations may play a role in determining tumourigenicity and metastatic potential as well as conferring sensitivity to EGFR inhibition. Further investigation into how EGFR interacts with other oncogenic pathways will be important to aid the understanding and treatment of breast cancer. For example, treatment of ectopic mammary fat pad tumours in mice with a combination of chemotherapy, and PARP inhibitor and EGFR-targeted radioimmunotherapy was significantly more effective in preventing and even reversing tumour growth than single or double agent treatments [35].

A recent paper has described the resistance or sensitivity of various MCF10A mutant isogenic lines to EGFR, PI3K and mTOR inhibitors, both alone and in combination [96]. The isogenic mutations include the H1047R mutation in PIK3CA and the E746-A750 deletion in EGFR. Both of these isogenic lines demonstrated similar effects in response to gefitinib as seen in our assays with a general increase in sensitivity to the EGFR inhibitors. However, there are differences, including in the scale of the effects that may be accounted for the different models used. In our study the MCF10A lines overexpressed EGFR mutants while the isogenic lines used by Glaysher et al [96] were heterozygous and under normal promoter activity. Also, the MCF10CA1a lines used in our study have both the H1047R PIK3CA mutation and EGFR mutant overexpression. As isogenic activating mutations in PIK3CA also increase MCF10A cell sensitivity to EGFR inhibitors, this may explain the enhanced sensitivity to EGFR inhibitors exhibited by the parental and overexpression MCF10CA1a lines.

In conclusion, mutant *EGFR* can enhance some of the tumourigenic properties in the MCF10A/MCF10CA1a breast cancer progression model including proliferation, migration, invasion and tumour formation. Unlike MCF10A cells, MCF10CA1a cells did not exhibit increased sensitivity to gefitinib when expressing mutant *EGFR* suggesting that genetic context is imporant in determining responsiveness to EGFR inhibition. This may be due to copy number alterations or somatic mutations that occurred in the evolution of MCF10A to MCF10CA1a. Exome sequencing revealed a number of altered genes implicated in cancer that are specific to the MCF10CA1a line and which may represent alterations which, in addition to the mutation in *PIK3CA*, are required to impart tumourigenic properties upon the MCF10A line. These genes could be investigated further for their involvement in resistance to EGFR inhibition in breast cancer, and in breast cancer in general.

## Supporting Information

S1 Fig Protein expression of EGFR in overexpressing cell lines. EGFR expression was assessed by immunoblotting with anti-EGFR antibody in MCF10A **(A)** and MCF10CA1a **(B)** cell lines expressing EGFR wild-type (WT), EGFR-G719S (GS) or EGFR-E746-A750del (Del) (10A: MCF10A, CA: MCF10CA1a-EV). Equivalent loading was verified by immunoblotting with either β-actin or α-tubulin, as indicated.
(TIF)

S2 Fig EGFR expression in MCF10A cells does not affect cell morphology in 2D culture. Cells were cultured in medium containing 20 ng/mL EGF and subconfluent cultures were imaged in random fields of view. Image contrasts was enhanced and equalized for visualization purposes. Scale bars = 100 μm.
(TIF)

S3 Fig Spheroids formed by EGFR expressing MCF10A cells have partially occluded luminal spaces. Cells were cultured in the presence (5 ng/mL) or absence of EGF in growth factor free Matrigel containing media for 14 days. MCF10A and MCF10A EGFR-WT do not form spheres in the absence of EGF. Spheroids were fixed and stained for DAPI (blue) and α-tubulin (green) and examined by confocal microscopy. Images are sequential series of Z-stack images with numbers to indicate stack distance relative to the first image in each set. Scale bars = 100 μm. Contrasts were enhanced for visualization purposes.
(TIF)

S4 Fig Transient distant site colonization from MCF10CA1a-*EGFR*-DEL-induced primary tumours after resection does not lead to true metastasis formation in adult female BALB/c nude mice. MCF10CA1a -*EGFR*-DEL-induced primary tumours from the mammary fat pad were surgically removed once tumour volume reached 250mm^3^ and mice were followed up by BLI and imaged at the indicated times post injection. Luciferase signals were observed outside the primary tumour site for up to 6 months but eventually disappeared.
(TIF)

S5 Fig Dose response to EGFR inhibitors. (**A-D**) Cells were cultured in the absence (- EGF, **A**, **B**) or presence (+ EGF, **C**, **D**) of EGF, treated with the indicated dose of gefitinib (Gef) for 48 hours and survival assessed by monitoring changes in confluency (**A**, **C**) or cell death (**B**, **D**) using real-time imaging on the Incucyte Zoom. **(E**-**F)** Cells were cultured in EGF-free media, treated with the indicated dose of gefitinib (Gef), afatinib (Afat), or cetuximab (Cet) for seven days and cell death measured using the MTS assay. Shown are data representative of the results from two to four independent experiments. CA: MCF10CA1a-EV, WT: MCF10CA1a-*EGFR*-WT, GS: MCF10CA1a-*EGFR*-GS, DEL: MCF10CA1a-*EGFR*-DEL.
(TIF)

S6 Fig Dose response curves to EGFR inhibitors combined with chemotherapy. Cells were cultured in the EGF-free media, treated with the indicated dose of docetaxel/ doxorubicin (5:1 ratio docetaxel:doxorubicin) either alone (DD), or with the half IC50 dose of gefitinib (DDG) or afatinib (DDA) for seven days and survival measured using the MTS assay. **A.** MCF10CA1a-EV. **B.** MCF10CA1a-*EGFR*-WT. **C.** MCF10CA1a-*EGFR*-GS. **D.** MCF10CA1a-*EGFR*-DEL. Data shown are representative of the results from three independent experiments.
(TIF)

S1 Table Regions of copy number variation in MCF10A cells. (XLSX)

S2 Table Regions of copy number variation in MCF10CA1a cells. (XLSX)

S3 Table Summary of genes with copy number variations in MCF10A and MCF10CA1a. (XLSX)

S4 Table Single nucleotide variations and INDELs in the genome of MCF10CA1a. (XLSX)

S5 Table Single nucleotide variations and INDELs in the genome of MCF10A. (XLSX)

S6 Table Frequency of genes with potentially damaging* rare variants in MCF10CA1a are altered** in breast cancer in The Cancer Genome Atlas (TCGA) dataset. These results are based upon data generated by the TCGA Research Network: http://cancergenome.nih.gov/. * Potentially damaging mutations include missense mutations with predicted damaging effects, nonssense mutations and out-of-frame indels. ** Alterations are the total of the copy number variations, somatic mutations and gene expression changes reported in the TCGA dataset. Total number of tumours in dataset = 1061.
(XLSX)

S7 Table Top diseases and molecular functions for the gene list of MCF10A and MCF10CA damaging variants from Ingenuity Pathway Analysis. (XLSX)
