# Supplementary figures and images for: Using the MCF10A/MCF10CA1a Breast Cancer Progression Cell Line Model to Investigate the Effect of Active, Mutant Forms of EGFR in Breast Cancer Development and Treatment Using Gefitinib
Source: PLoS One. 2015 May 13;10(5):e0125232. doi: 10.1371/journal.pone.0125232 (PMC4430383; doi:10.1371/journal.pone.0125232)

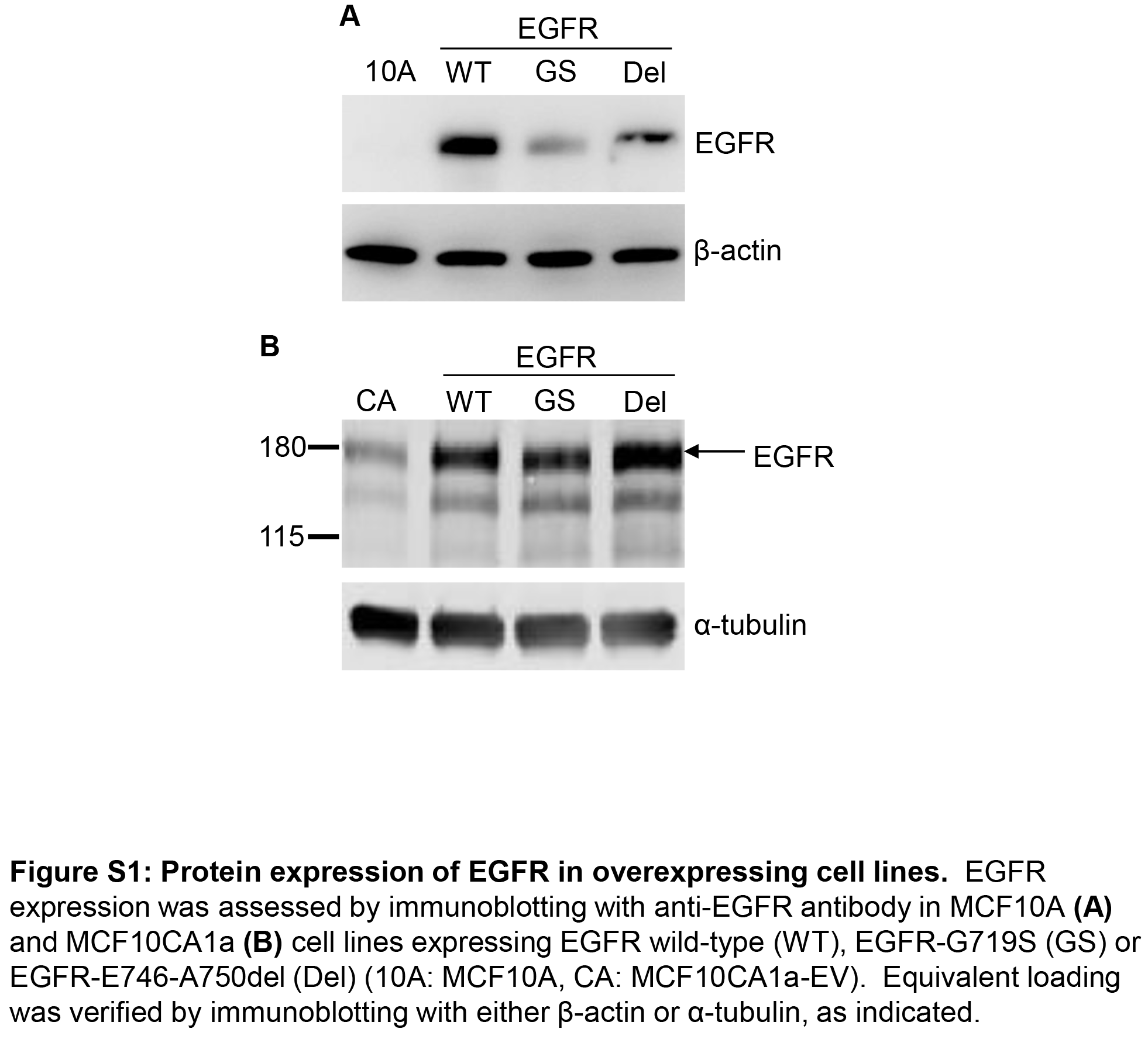

Supplement: S1 Fig — EGFR expression was assessed by immunoblotting with anti-EGFR antibody in MCF10A (A) and MCF10CA1a (B) cell lines expressing EGFR wild-type (WT), EGFR-G719S (GS) or EGFR-E746-A750del (Del) (10A: MCF10A, CA: MCF10CA1a-EV). Equivalent loading was verified by immunoblotting with either β-actin or α-tubulin, as indicated. (TIF) [file pone.0125232.s001.tif]

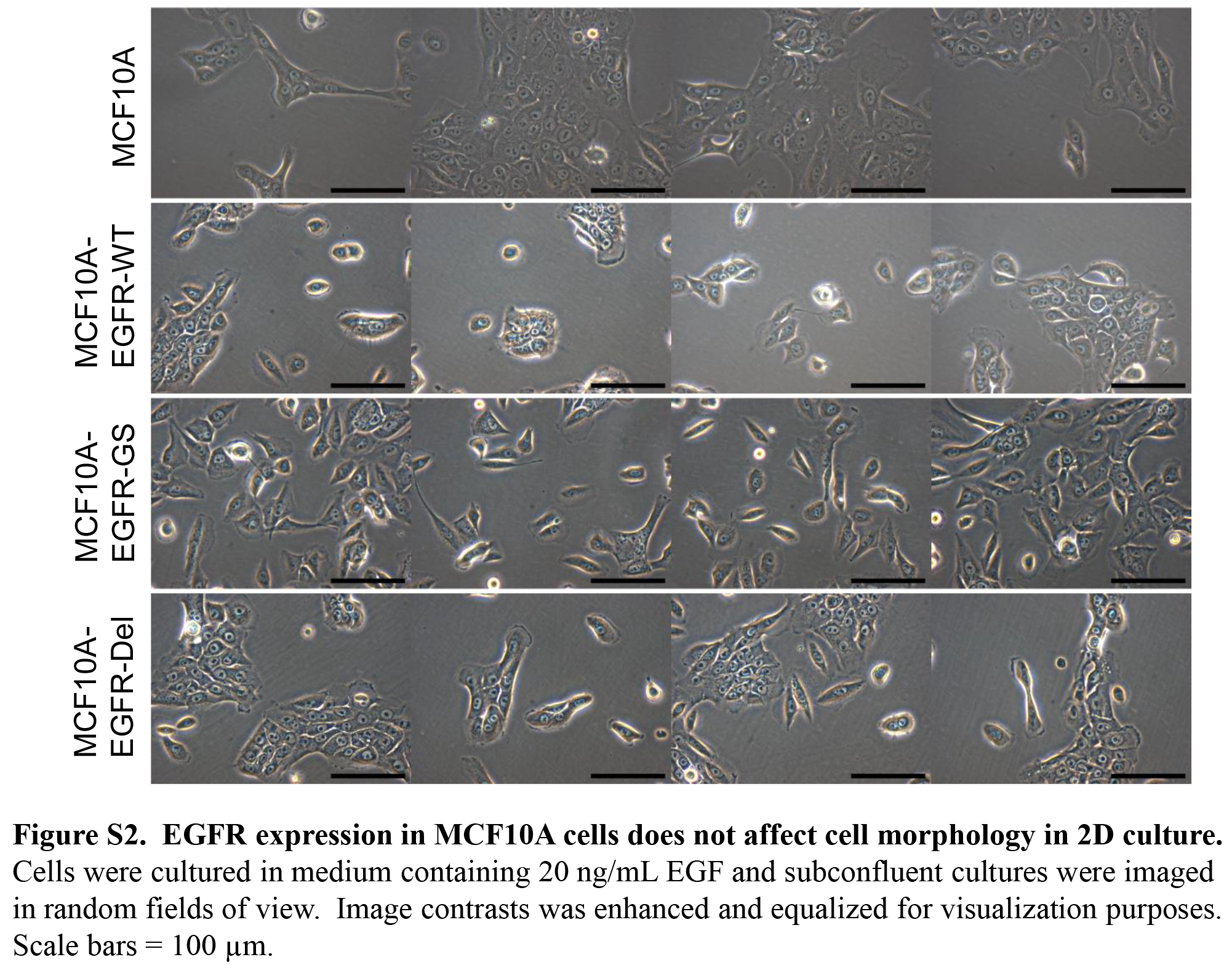

Supplement: S2 Fig — Cells were cultured in medium containing 20 ng/mL EGF and subconfluent cultures were imaged in random fields of view. Image contrasts was enhanced and equalized for visualization purposes. Scale bars = 100 μm. (TIF) [file pone.0125232.s002.tif]

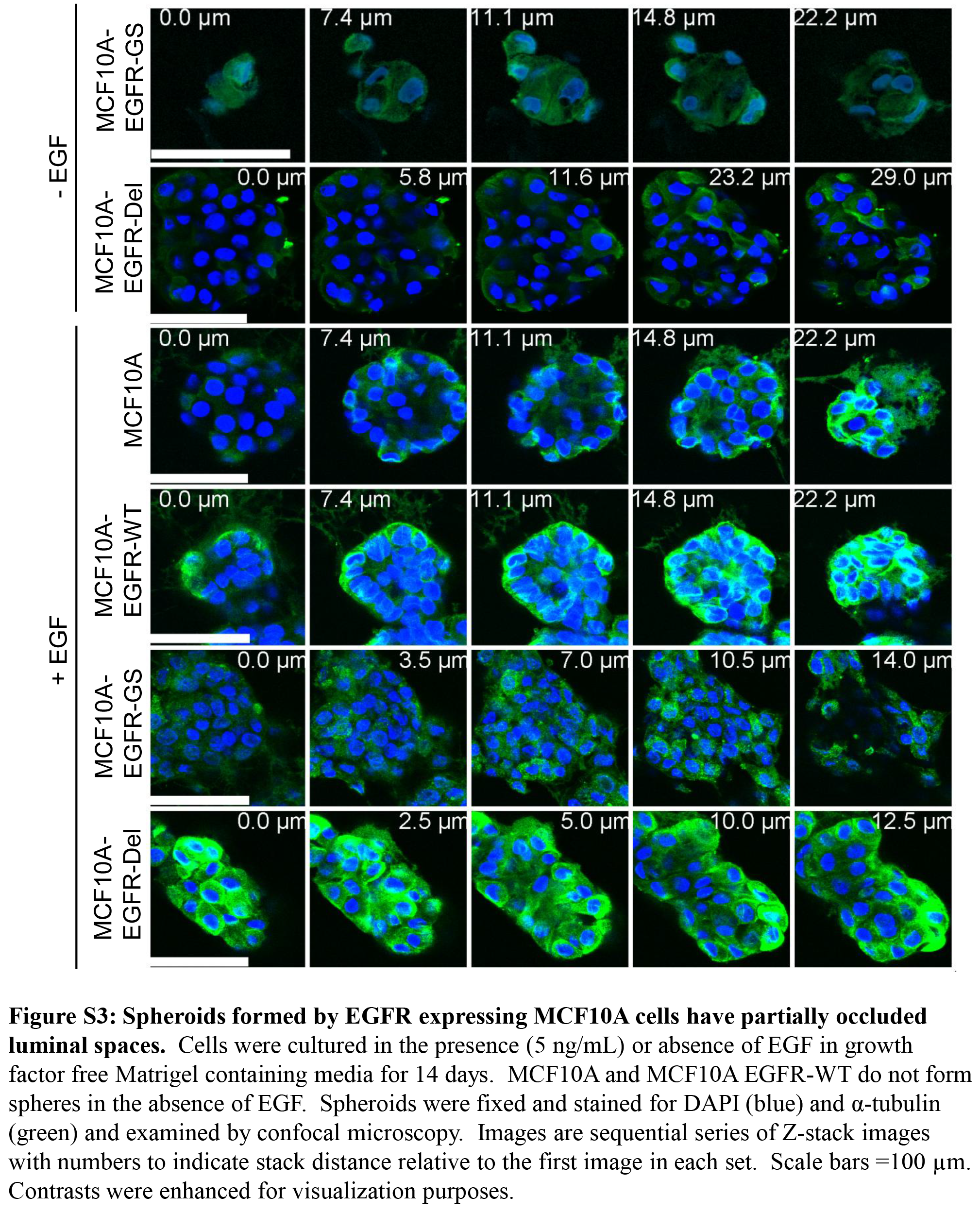

Supplement: S3 Fig — Cells were cultured in the presence (5 ng/mL) or absence of EGF in growth factor free Matrigel containing media for 14 days. MCF10A and MCF10A EGFR-WT do not form spheres in the absence of EGF. Spheroids were fixed and stained for DAPI (blue) and α-tubulin (green) and examined by confocal microscopy. Images are sequential series of Z-stack images with numbers to indicate stack distance relative to the first image in each set. Scale bars = 100 μm. Contrasts were enhanced for visualization purposes. (TIF) [file pone.0125232.s003.tif]

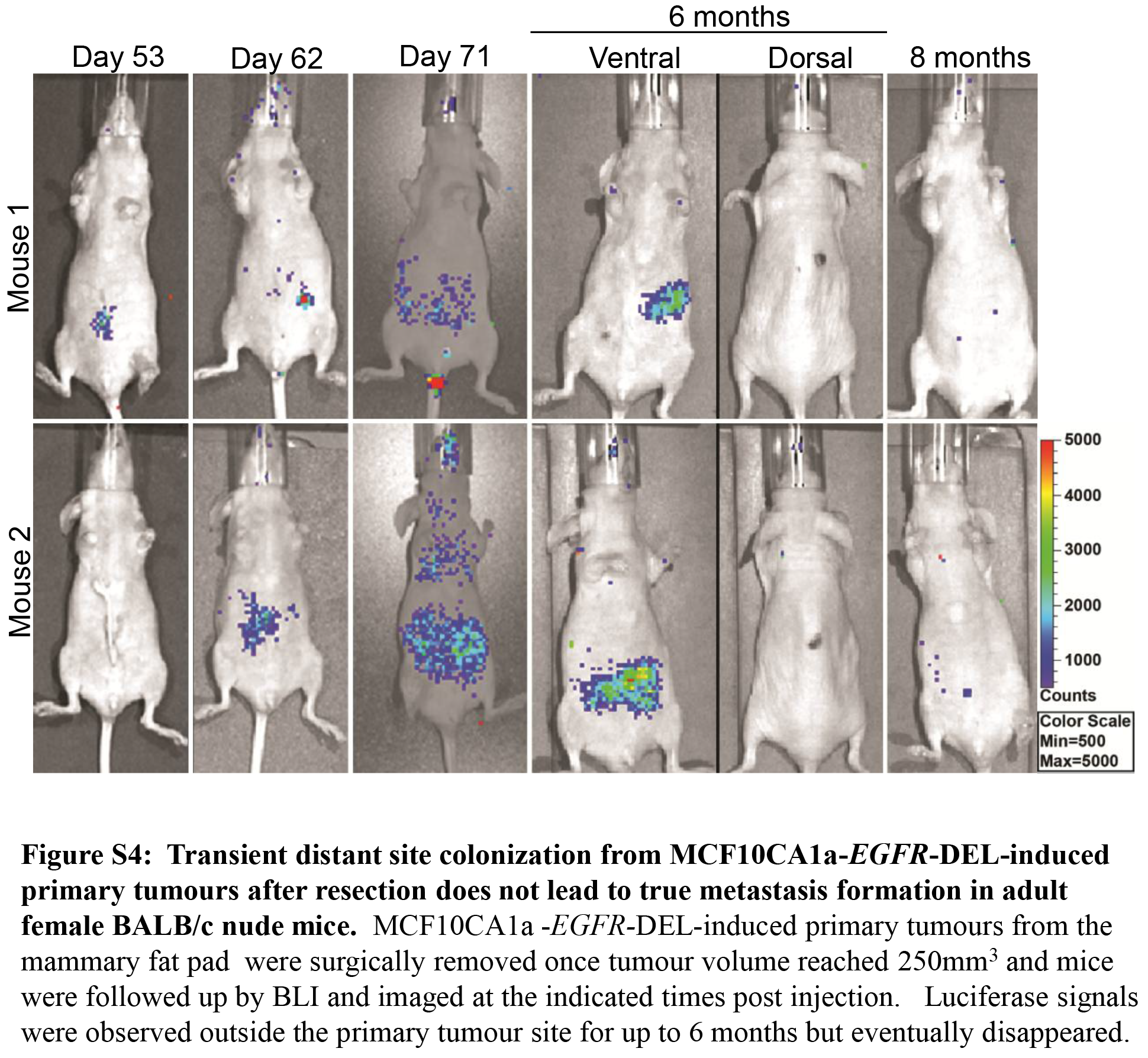

Supplement: S4 Fig — MCF10CA1a -EGFR-DEL-induced primary tumours from the mammary fat pad were surgically removed once tumour volume reached 250mm3 and mice were followed up by BLI and imaged at the indicated times post injection. Luciferase signals were observed outside the primary tumour site for up to 6 months but eventually disappeared. (TIF) [file pone.0125232.s004.tif]

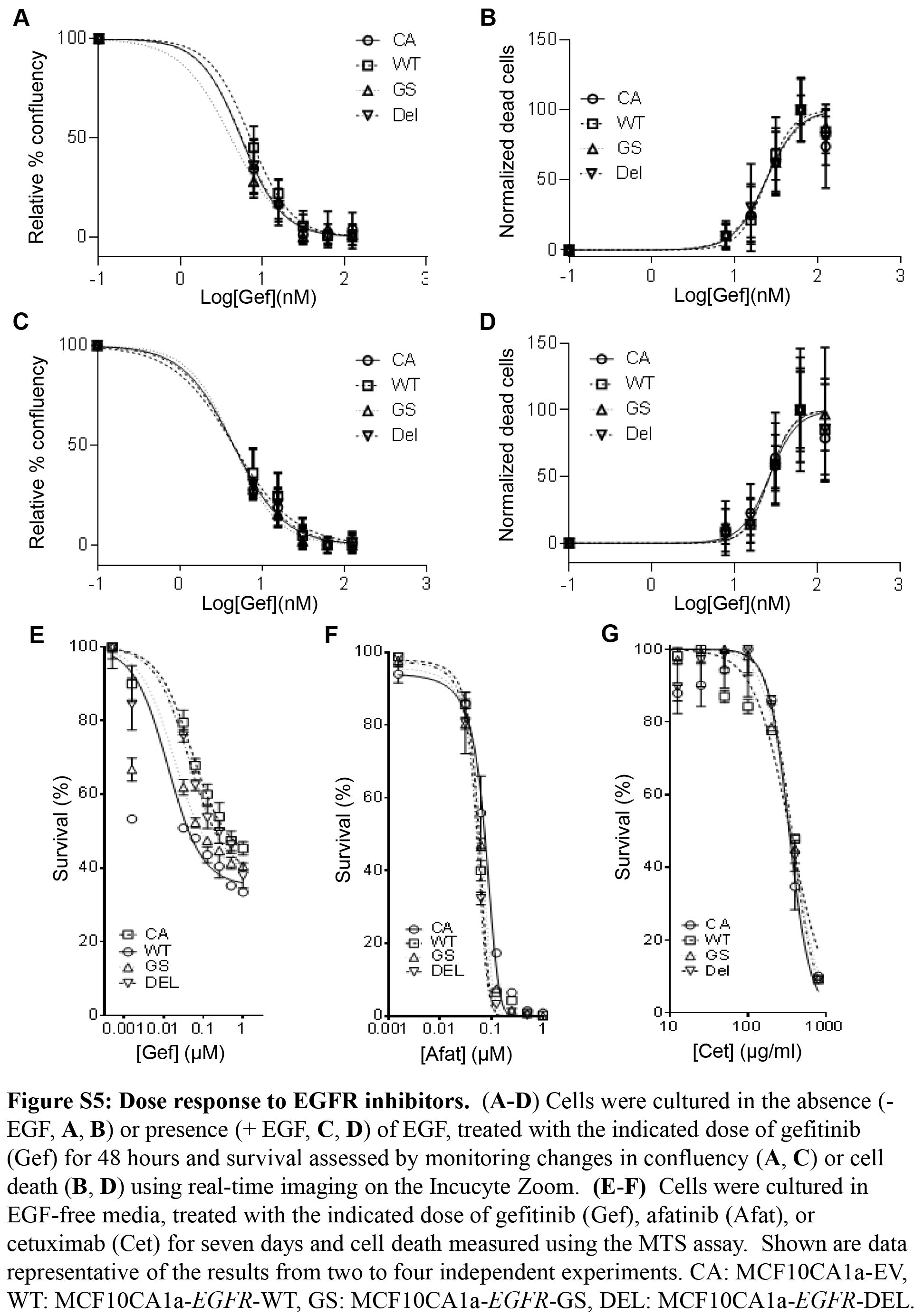

Supplement: S5 Fig — (A-D) Cells were cultured in the absence (- EGF, A, B) or presence (+ EGF, C, D) of EGF, treated with the indicated dose of gefitinib (Gef) for 48 hours and survival assessed by monitoring changes in confluency (A, C) or cell death (B, D) using real-time imaging on the Incucyte Zoom. (E-F) Cells were cultured in EGF-free media, treated with the indicated dose of gefitinib (Gef), afatinib (Afat), or cetuximab (Cet) for seven days and cell death measured using the MTS assay. Shown are data representative of the results from two to four independent experiments. CA: MCF10CA1a-EV, WT: MCF10CA1a-EGFR-WT, GS: MCF10CA1a-EGFR-GS, DEL: MCF10CA1a-EGFR-DEL. (TIF) [file pone.0125232.s005.tif]

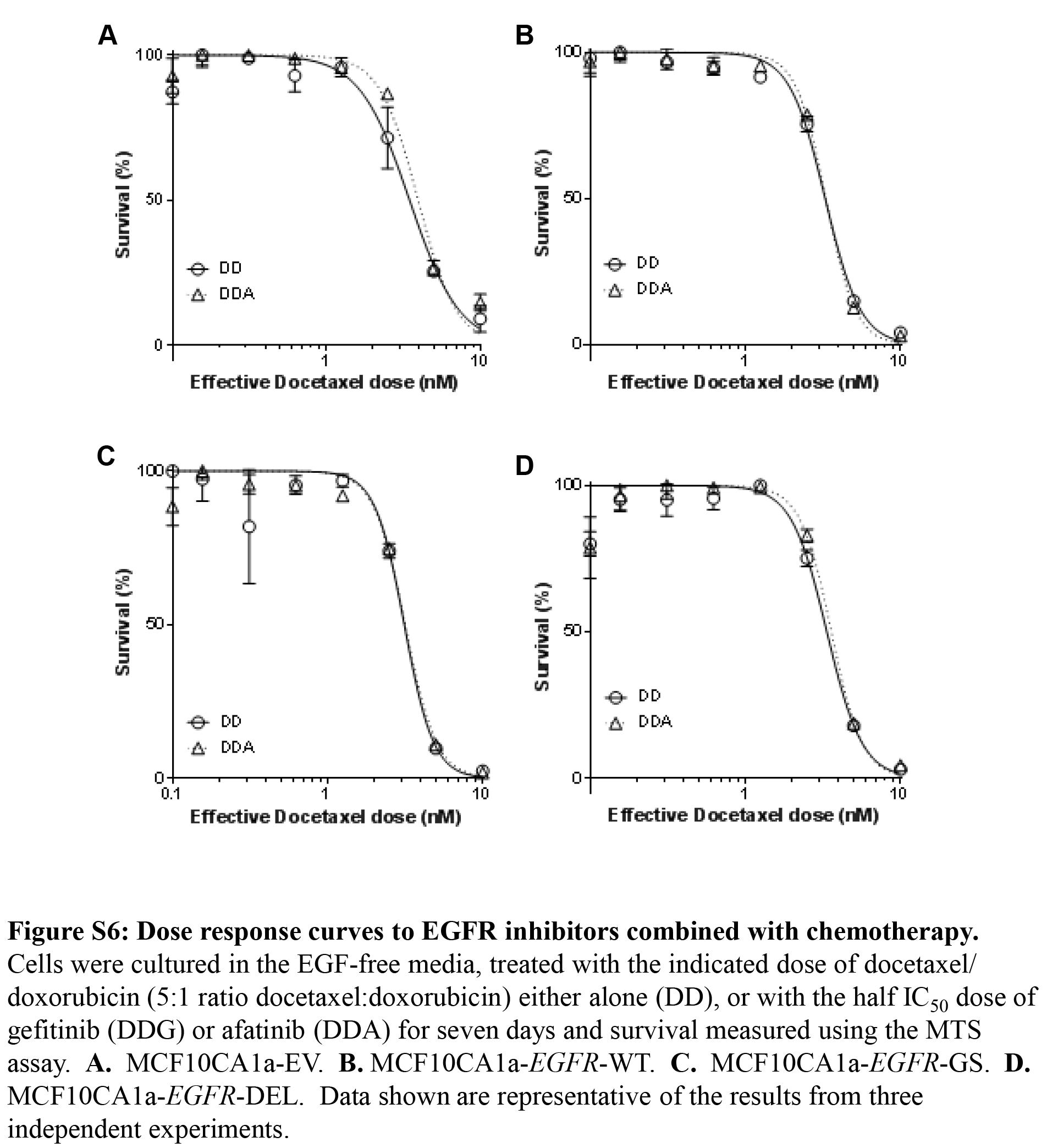

Supplement: S6 Fig — Cells were cultured in the EGF-free media, treated with the indicated dose of docetaxel/ doxorubicin (5:1 ratio docetaxel:doxorubicin) either alone (DD), or with the half IC50 dose of gefitinib (DDG) or afatinib (DDA) for seven days and survival measured using the MTS assay. A. MCF10CA1a-EV. B. MCF10CA1a-EGFR-WT. C. MCF10CA1a-EGFR-GS. D. MCF10CA1a-EGFR-DEL. Data shown are representative of the results from three independent experiments. (TIF) [file pone.0125232.s006.tif]
